# Preprocessing Large-Scale Conversational Datasets: A Framework and Its Application to Behavioral Health Transcripts

**DOI:** 10.2196/78082

**Published:** 2025-10-24

**Authors:** Paz Mor Naim, Shiri Sadeh-Sharvit, Samuel Jefroykin, Eddie Silber, Dennis P Morrison, Ariel Goldstein

**Affiliations:** 1 Department of Cognitive and Brain Sciences, Hebrew University of Jerusalem, Mount Scopus, Jerusalem, 9190500, Israel, 972 025882888; 2 Eleos Health, Waltham, MA, United States; 3 Palo Alto University, Palo Alto, CA, United States; 4 Morrison Consulting, Bloomington, IN, United States; 5 Business School, Hebrew University of Jerusalem, Jerusalem, Israel; 6 Department of Psychology, Azrieli Israel Center for Addiction and Mental Health (Azrieli ICAMH), Hebrew University of Jerusalem, Jerusalem, Israel

**Keywords:** artificial intelligence, behavioral health, clinical documentation, clinical texts, conversational transcripts, data preprocessing, data quality assessment, health informatics, health information systems, large language models, natural language processing, psychotherapy, text classification

## Abstract

**Background:**

The rise of artificial intelligence and accessible audio equipment has led to a proliferation of recorded conversation transcripts datasets across various fields. However, automatic mass recording and transcription often produce noisy, unstructured data that contain unintended recordings such as hallway conversations, media (eg, TV, radio), or transcription inaccuracies as speaker misattribution or misidentified words. As a result, large conversational transcript datasets require careful preprocessing and filtering to ensure their research utility. This challenge is particularly relevant in behavioral health contexts (eg, therapy, counseling) where deriving meaningful insights, specifically dynamic processes, depends on accurate conversation representation.

**Objective:**

We present a framework for preprocessing large datasets of conversational transcripts and filtering out *non-sessions*—transcripts that do not reflect a behavioral treatment session but instead capture unrelated conversations or background noise. This framework is applied to a large dataset of behavioral health transcripts from community mental health clinics across the United States.

**Methods:**

Our approach integrated basic feature extraction, human annotation, and advanced applications of large language models (LLMs). We began by mapping transcription errors and assessing the number of non-sessions. Next, we extracted statistical and structural features to characterize transcripts and detect outliers. Notably, we used LLM perplexity as a measure of comprehensibility to assess transcript noise levels. Finally, we used zero-shot prompting with an LLM to classify transcripts as sessions or non-sessions, validating its output against expert annotations. Throughout, we prioritized data security by selecting tools that preserve anonymity and minimize the risk of data breaches.

**Results:**

Initial assessment revealed that transcription errors—such as incomprehensible segments, unusually short transcripts, and speaker diarization issues—were present in approximately one-third (n=36 out of 100) of a manually reviewed sample. Statistical outliers revealed that high speaking rate (>3.5 words per second) is associated with short transcripts and answering machine messages, while short conversation duration (<15 min) was an indicator for case management sessions. The 75th percentile of LLM perplexity scores was significantly higher in non-sessions than sessions (permutation test mean difference = −258, *P* =.02), although this feature alone offered only moderate classification performance (precision =0.63, recall =0.23 after outlier removal). In contrast, zero-shot LLM prompting effectively distinguished sessions from non-sessions with high agreement to expert ratings (κ=0.71) while also capturing the nature of the meeting.

**Conclusions:**

This study’s hybrid approach effectively characterizes errors, evaluates content, and distinguishes text types within unstructured conversational dataset. It provides a foundation for research on conversational data, key methods, and practical guidelines that serve as crucial first steps in ensuring data quality and usability, particularly in the context of mental health sessions. We highlight the importance of integrating clinical experts with artificial intelligence tools while prioritizing data security throughout the process.

## Introduction

As one of our primary communication tools, conversations offer a window into human relationships. Speaking with each other is one of the fundamental aspects of our existence. Consequently, transcripts of conversations have garnered significant interest in research across various disciplines [1]. Analyzing conversational data is a central research focus in fields such as health care [2,3], law [4], customer support [5], negotiations [6], education [7], and behavioral treatment and psychotherapy [8-10].

Among these diverse domains, talk therapy, which relies on a conversation between 2 or more individuals, is a particularly intriguing case for conversation analysis. Analysis of treatment sessions can enhance our understanding of different therapeutic protocols [9,11-14]; improve clinical training [15-17]; and provide insights into the relationships between conversational elements, therapeutic outcomes, and the therapeutic alliances [8,10,18,19]. Conversation analysis research of behavioral health sessions can provide comprehensive insights into the intricate dynamics of therapeutic interactions [2]. By meticulously examining communicative choices, researchers can understand how specific counseling strategies impact client engagement, identify successful intervention techniques, and reveal nonlinear patterns of change within therapy sessions [20,21]. This research methodology allows professionals to study the dyadic or group psychotherapeutic processes, predict potential treatment outcomes, develop more effective interventions, and ultimately enhance the quality of behavioral health care [14,22].

Historically, collecting, transcribing, and analyzing treatment conversations have required significant human effort. This is now changing, thanks to 2 major technological advances. First, recent developments in audio capturing technologies and the ubiquitousness of smart devices simplify the collection of conversation data. Second, the abundance of artificial intelligence (AI)–based speech-to-text tools [23-25] has automated transcription and speaker recognition tasks. These innovations have made it possible to capture high-quality human speech in diverse settings with less manual effort [26]. Consequently, large-scale transcription of audio files is now feasible with unprecedented speed and precision. The automation of these processes introduces new challenges. Transcripts’ quality depends on several factors: the recording devices and their placement in the room, background noise, internet connection stability, and accurate speech-to-text and speaker diarization models. Additionally, characteristics of the conversation itself, such as slurred speech, dialects, rare languages, or use of slang, all pose further challenges for automatic speech recognition (ASR) models [27]. Similarly, interrupted, rapid, or overlapping speech, unknown number of speakers as well as same-sex speakers [28,29] each introduce complications for speaker recognition systems.

Moreover, the expected growth in large datasets accompanying this automation amplifies these challenges. Large and diverse datasets might include recordings of different quality levels and therefore are prone to various types of errors. Furthermore, when recordings are made routinely and automatically, unintentional recordings—irrelevant conversations, phone calls, empty moments, or accidental noise—may occur. This illustrates how large volumes of automatically generated transcripts are unstructured and susceptible to errors. Therefore, guidelines and methods for error handling, assessing, and filtering should be established.

These challenges are well-known in the broader field of health care, which has long grappled with extracting meaningful information from unstructured data such as clinical notes, discharge summaries, or patient-generated text [30,31]. As the field has evolved, various preprocessing pipelines and methodological frameworks have been proposed for handling missing metadata, variable data quality, and inconsistent documentation practices [32-34]. However, most existing work focuses on clinical records, and less attention has been given to large-scale, conversational health care data.

Yeomans et al [35] have recently proposed a methodological pipeline for building conversation-based research, starting with the planning and collection of conversations and going through editing and analyzing. However, with the availability of ambient AI and larger datasets, many researchers may be working with secondary data—datasets they have not collected themselves. Therefore, they might not have access to the original recordings, to the speech-to-text models, nor to the transcripts editing process. Lacking a ground truth for verification necessitates expanding these guidelines to address such challenges.

In the case of behavioral treatment sessions, the noisiness of the transcript and its accurate representation of the conversation are key for detecting the interventions applied and extracting treatment insights [25,36]. For instance, misidentifying speakers could compromise insights regarding the therapeutic alliance [18,21,37]. Understanding which conversations are professional and which are accidental recordings is the first step in this endeavor.

In this paper the abovementioned challenges are addressed, and a methodological approach for preprocessing a large dataset of behavioral treatment transcripts without access to their respective original recordings is presented. First, a systematic approach for characterizing the data is outlined, followed by methods that allow its assessment for future analysis. This methodology is then illustrated by applying it to a large dataset of deidentified behavioral health sessions. Finally, the strengths and limitations of our approach are discussed. We hope to promote the integration of computational tools in traditional talk therapy and to offer relevant methods for any dataset of conversational transcripts.

## Methods

### Data and Settings

We analyzed 22,337 behavioral treatment sessions from 50 behavioral health programs across the United States collected through the Eleos Health platform between June 2020 and January 2024. Eleos Health’s digital platform is designed to promote behavioral treatment quality by providing intervention feedback, supporting clinical decision-making, and enabling progress note automation. Sessions were processed as part of the routine implementation of ambient AI tools within participating behavioral health programs. All sessions underwent transcription, deidentification, and anonymization using Eleos’ proprietary models before inclusion in the study. The research team had no access to audio recordings or transcripts prior to this process, and only deidentified data were analyzed. Processed transcripts were stored as comma-separated values files with 3 primary columns: deidentified speaker labels, timestamps, and content. Each content row contained a speech segment from either the therapist or the client—typically up to several sentences—segmented by the Eleos ASR model. Session metadata included random therapist and client ID numbers, organization name, session date, and treatment delivery method (phone, video conferencing, or in-person). This study was conducted in concert with the STROBE Checklist [38].

### Ethical Considerations

This study was determined exempt from review by the Sterling Institutional Review Board under the Department of Health and Human Services Exemption Category 4. This exemption permits secondary research using identifiable health information when the data are either publicly available or recorded in such a way that subjects cannot be reidentified, and there is no direct interaction with participants. All research procedures adhered to applicable ethical guidelines and regulations. Clients and therapists provided informed consent for the use of anonymized, deidentified session data in secondary research conducted by the company. Both parties retained the option to opt out of having their sessions processed. All data used in this study were deidentified prior to analysis, and no identifiable information was accessed by the research team. No compensation was provided for participation in this secondary analysis, and no images or supplementary materials contain identifiable individuals.

### Data Analysis Approach

**Figure 1.**
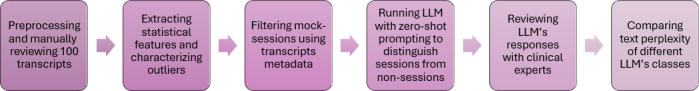
Work pipeline—starting with manual review of a dataset subset, followed by statistical feature extraction and outlier detection. Next, we apply zero-shot prompting with a large language model (LLM) to classify transcripts as sessions or non-sessions and validate the LLM’s decisions against human annotators. Finally, we compare LLM’s perplexity of different classes.

Our data analysis approach consisted of several stages, each employing different methods for characterizing and filtering the dataset (see Figure 1). The initial stages were exploratory, beginning with a manual review to assess data’s content and quality. This process helped us identify categories of files and potential errors in the data, enhancing our understanding of the dataset and the features most suitable for characterization and filtering. Subsequent steps focused on directly classifying transcripts as “sessions” or “non-sessions” (eg, nonprofessional conversations, noise, mock sessions). Using available metadata, we filtered out mock sessions (non-sessions) that had known identifiers. Additionally, we applied zero-shot prompting with a large language model (LLM) to distinguish therapy sessions from irrelevant conversations. Automation played a critical initial role in this phase; however, human expertise - specifically trained psychologists - was incorporated to validate the automated classifications. At this stage, aligned with our primary goal of preparing the dataset for psychological research, various types of interventions were distinguished, and the sessions category was restricted only to formal treatments (excluding peer support, sponsorship, and case management). Notably, we did not prompt the model with these exclusion criteria, testing its ability to make such distinction based solely on therapeutic elements.

#### Dataset Preprocessing

##### Dataset Characteristics

We identified potential duplicates in the dataset by analyzing the similarity between file names. This method is efficient in terms of processing time and computational resources, as it avoids comparing entire text contents. The underlying assumption is that identical files might be mistakenly saved under similar file names. To measure similarity, we used Python’s SequenceMatcher function [39]. File names were considered similar if the ratio of their longest matching segment to the total number of characters exceeded 75%. Files meeting this threshold were then manually reviewed to confirm duplication.

Additionally, some transcripts were recorded in languages other than English. To detect the languages, we used the langdetect library in Python [40].

##### Initial Assessment

A total of 100 randomly selected transcripts were manually reviewed by human raters, with 12 segments analyzed from each transcript to identify common errors and quantify their prevalence. At least 4 categories of errors were defined: (1) non-sessions: transcripts that clearly did not represent a session. At this stage, no distinction was made between types of intervention (eg, peer support, sponsorship, or formal treatment); (2) too short: transcripts with a total duration of less than 2700 seconds (45 min, which approximates the expected session length); (3) unreadable: transcripts with excessive missing words, duplicated segments, or incomprehensible text—readers could not infer the missing content or the meaning of the segment. For example, a transcript with multiple repeated filler phrases (eg, “uh-huh, uh-huh, uh-huh…”) was generally deemed unreadable; (4) speaker diarization errors: transcripts with substantial speaker attribution mistakes.

In some cases, annotators indicated that additional context was required to ascertain the presence of an error.

### Features Learning

We collected statistics about the whole dataset and each session which included (1) conversation length, (2) speaking rate, (3) frequent words, and (4) segment perplexity.

In addition to characterizing the dataset, these features serve as potential indicators for assessing the data’s compatibility for further analysis. For instance, they can highlight recording errors or determine whether the content is session related. In the following section, we elaborate on some of these features and explain how they were calculated for each session.

#### Conversation Length

Conversation length was calculated by extracting the end timestamp of the final speech segment in the transcript. This method does not account for any time elapsed between when the recording device was activated and the conversation began. However, if the session starts with silence, this approach accurately reflects the length of the conversation.

#### Speaking Rate

Speaking rate, defined as the average number of words spoken per second, has been employed to predict speaker characteristics [41], detect speech anomalies, and identify changes in conversational dynamics or context [42]. In American conversational speech, the reported average speaking rate ranges from 1.85 to 4.86 words per second (WPS) [41]. Values outside this range may indicate abnormal recordings, such as background noise or transcription errors.

For each transcript, speaking rate was calculated by averaging across segment-level speaking rates. Because silent pauses within segments were not available, we could not adjust for them as is commonly done. However, segments were brief (typically a few seconds) and cut before long silences, making the absence of silent moments negligible and this measure a reasonable approximation. For comparison, we also calculated a global speaking rate by dividing the total word count by the overall conversation length, which incorporates silent periods.

#### Word Frequency

We explored the content expressed in the transcripts by extracting the most common nouns in the dataset. First, we cleaned each text by removing stop words [43] and technical terms (a list of which can be found in Multimedia Appendix 1). These filtered words were typically used in bureaucratic contexts, such as scheduling meetings or filling out professional forms. Next, we separated each transcript into 2 speaker partitions: therapist and client. Using Python’s library, Spacy [44], we collected all the nouns spoken across all transcripts for each speaker. This content analysis revealed common topics discussed during conversations. Additionally, unexpected words that emerged may indicate common errors made by the ASR model.

#### Perplexity

Perplexity is a measure of a text sequence, indicating its likelihood of being produced by a language model [45]. It is the model’s loss when given the words in the sequence and is conceptually similar to the cross-entropy between the model’s and the sequence’s probability distribution:

,$$Perplexity\left(segment\right)=exp\left(-\frac{1}{N}\sum_{i=1}^{N}log\left(P\left(w_{i}|w_{i-1,...,}w_{1}\right)\right)\right)$$

where *w*_*i*_ is a token in the segment’s sequence. Due to the short length of segments, the number of tokens is typically smaller than the model’s context length. Consequently, the probability of *w*_*i*_ is calculated considering all previous tokens in the sequence. High perplexity suggests that the model is less likely to generate such a segment.

We used OpenAI’s *GPT-2* (locally, through the *Hugging Face* platform [46,47]) to compute the perplexity of approximately 5 million segments in 9067 transcripts. Perplexity was calculated at the segment level and then aggregated within each transcript. We hypothesized that higher perplexity would reflect higher semantic complexity, thereby acting as a marker for actual sessions. However, we also anticipated that extreme values of perplexity could indicate recording errors or corrupted transcripts that do not represent a therapeutic conversation. Thus, we propose that therapeutic conversations will generally have higher average perplexities but will not show the highest maximum values. To test this assumption, we extracted the average perplexity, standard deviation of perplexity, and maximal perplexity for each transcript. Additionally, to get an accurate representation of the upper-bound perplexity values without being over-influenced by outliers, we calculated the 75th percentile of perplexity values for each transcript.

### Classification—Distinguishing Sessions From Conversations

#### Model and Platform

To classify transcripts of behavioral treatment to sessions and non-sessions, we queried an LLM with a zero-shot approach. We used the Amazon Bedrock platform [48] that enables running closed models through a third party, ensuring that the data were not shared or exposed to the model’s provider. The model selection out of the available platform’s options was based on criteria of cost, context length, and proven reliability. Table S1 in Multimedia Appendix 2 presents the cost of different tokens at the time of writing. Anthropic’s Claude [49] was chosen because of its proficiency in human tasks and cost-efficiency [49-51]. Both the LLM and the platform were carefully selected to ensure data security. Amazon’s Bedrock platform served as a third party between the model and the user, enabling us to use the model non-locally without exposing our data. Data were not saved on the platform’s servers, nor were they used for the benefit of training the model [48,52].

#### Filtering and Subsetting

Based on the metadata, we excluded files from organizations that are not clinical public entities, such as academic institutes conducting mock sessions and trials (25% filtered). From this subset, we randomly selected 850 transcripts for automatic classification.

#### Classification With LLM

“Claude-3-Sonnet” was applied through Amazon’s Bedrock platform (model ID: “anthropic.claude-3-sonnet-20240229-v1:0”) with the following parameters: maximal generated words (max_out): 350; temperature: 0.3; and the remaining parameters were set to default. A detailed prompt with guidelines specifying the criteria for classifying a transcript as a session, and a format for providing a well explained answer were created to ensure the model performed the task as expected. In collaboration with an expert clinical psychologist, we compiled a list of elements that define a behavioral treatment session. The prompt addressed the following 5 core elements of a behavioral treatment session:

Dynamics: a correspondence where one of the sides shares experiences and the other focuses on listening and responding.

Content: the conversation contains personal matters, emotions and experiences, discussions about personal goals, thoughts, or behaviors.

Therapeutic elements: demonstration of active listening and empathy by one side, and use of therapeutic techniques such as reframing, providing coping strategies.

Professional language: therapeutic terminology, references for treatment plans, or previous sessions.

Context clues: mentions of confidentiality, session time limit, or scheduling future appointments.

The prompt specifically instructed the model to look for these elements in its decision-making process, explain its reasoning, and rate its certainty on a scale of 1-5. Additionally, we asked the model to provide a brief summary of the conversation content and to indicate whether it identified nontherapeutic conversational dynamics. These instructions were designed to ensure that the model addressed the entire conversation and understood its content. To maintain consistency, we used XML tags (see Multimedia Appendix 3).

#### Validation by Clinical Expert and Interrater Reliability

To validate the model’s classifications, 2 human raters independently classified 150 randomly selected transcripts. Both raters were graduate students in psychology or cognitive sciences, pursuing either clinical or theoretical training related to psychotherapy. Both were familiar with transcripts of behavioral health sessions prior to this task.

Raters were instructed to read transcript segments (approximately 12 turns) carefully and determine whether the segment belonged to an individual treatment session, excluding family or couple sessions, peer support, and case management calls. If a decision could not be made based on the provided segment, raters were encouraged to consult the full transcript.

These exclusion criteria were not part of the model’s automated classification process and may therefore have introduced some discrepancies between human and model decisions. Nevertheless, our aim was to assess whether applying instructions focused on contextual and relational dynamics of the conversation would naturally result in the exclusion of nonprofessional conversations. Interrater agreement was assessed using Cohen kappa and percent agreement. Cohen kappa higher than 0.61 is generally interpreted as a substantial agreement [53]. We then calculated the percent agreement of the human raters with the model for each category (session, non-session).

#### Perplexity of Sessions Versus Non-Sessions

To measure whether the 2 perplexity distributions can be assigned to 2 different distributions—sessions and non-sessions—we conducted a permutation test. Out of the transcripts for which we calculated segment perplexity, 335 transcripts were also assigned a class by the LLM: 285 sessions and 50 non-sessions.

We did not control the length of segments and their number, both are expected to affect the perplexity of a file. To ensure that the results are robust under filtering of extremely short transcripts and short segments, we calculated the same results after filtering segments with less than 5 or 10 words, and transcripts for which less than 10 or 20 segments. Finally, based on the results, we assessed the separability of sessions and non-sessions distributions by evaluating a perplexity-based classifier.

## Results

### Dataset Characteristics

#### Duplicates Identification and Language Detection

We identified 1 duplicate file and 1 empty file in the dataset. After removing these, the dataset consisted of 22,335 transcripts. The analysis revealed 18 different languages. The most common was English (98%), followed by Hebrew (0.7%).

#### Initial Assessment

The manual review of 100 transcripts yielded the following results: 46% of the transcripts were comprehensible and had *little to no errors*, 18% required a more thorough review for clear evaluation and 36% contained *clear errors*, which were categorized as follows (some transcripts had multiple error types): speaker identification errors (eg, confusing the therapist with the client, 42%); incomprehensible text (34%); too short to be indicative of the conversation type (22%); either non-session content or group sessions (11%) and *missing words or duplicated segments* (8%).

This preliminary evaluation found that the dataset was highly diverse, comprising the following transcript types:

1. Non-session content: The presence of non-session and group-session transcripts underscores the need for filtering mechanisms to exclude these from analysis.
2. Session quality: Based on this evaluation, we estimate that approximately half of the dataset contains sessions suitable for further analysis.
3. Speaker recognition errors: Given the frequency of speaker diarization errors, features that rely on speaker identification (eg, number of turns, spoken time by speaker) may be unreliable without corrections.
4. Incomprehensible text: This analysis underscores the importance of developing tools to improve text comprehensibility. With appropriate adjustments, more data could be rendered suitable for analysis.

### Features Learning

#### Conversation Length

**Figure 2.**
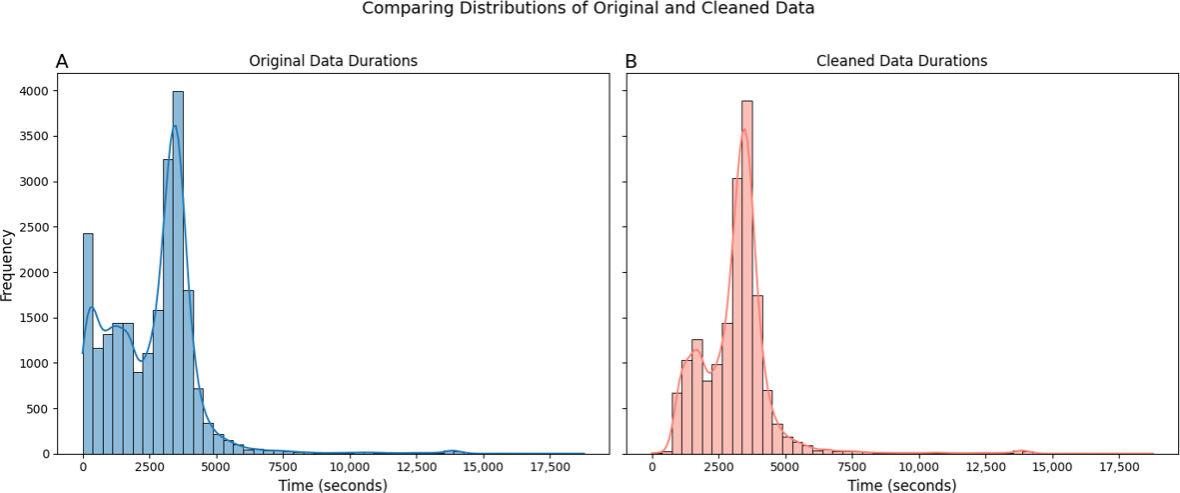
Comparison of transcript duration distributions before and after data cleaning. (**A) **Distribution of durations for all transcripts, including trial sessions and experimental recordings. (**B) **Distribution after excluding transcripts based on organization category metadata. The comparison highlights that many short-duration transcripts originated from irrelevant or excluded organizations.

**Figure 3.**
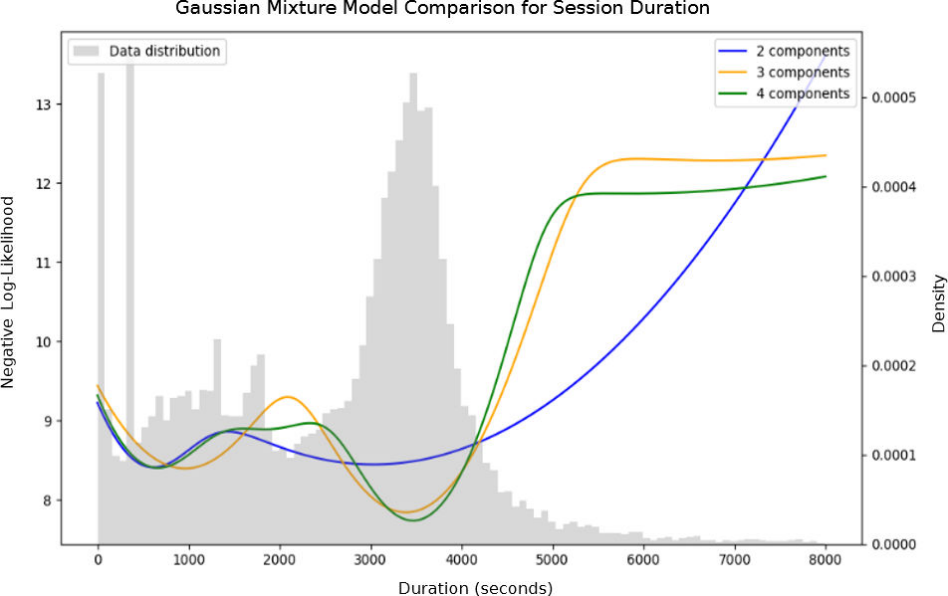
Comparison of Gaussian mixtures models (GMMs) with 2, 3, and 4 components fitted to the distribution of session durations (in seconds) based on transcript timestamps (Figure 2A). The negative log-likelihood is reported for each model, with lower values indicating better fit.

Conversations length ranged between 0.5 and 18,783 seconds, with an average of 2707 seconds and median of 3062 seconds. Figure 2 shows the full distribution of conversation length (A) and the same distribution after omitting conversations shorter than 15 minutes long (B). A Gaussian mixture model best fitted 3 Gaussian distributions for the full distribution and 2 for the reduced distribution. Figure 3 illustrates a comparison of the model’s full distribution fit for different numbers of components, and Table 1 presents the values of the Akaike information criterion and the Bayesian information criterion under each parameter.

**Table 1.** Akaike information criterion and Bayesian information criterion for different number of components computed for the Gaussian mixture models presented in Figure 3.

Components	AIC^a^	BIC^b^
2	39,3727	39,3767
3	38,4168	38,4232
4	38,3384	38,3473

a AIC: Akaike information criterion.

b BIC: Bayesian information criterion.

To support our findings, we examined the metadata to determine whether different conversation types (based on organization) were associated with different transcript length. Among the short transcripts (<15 min) that included conversational content, we found brief case management phone calls. The long transcripts (>1.2 h) included 2 sessions in a row, or a recording extended before or after a session. These patterns suggest the presence of 2 conversation populations, each with a different duration distribution.

#### Speaking Rate

**Figure 4.**
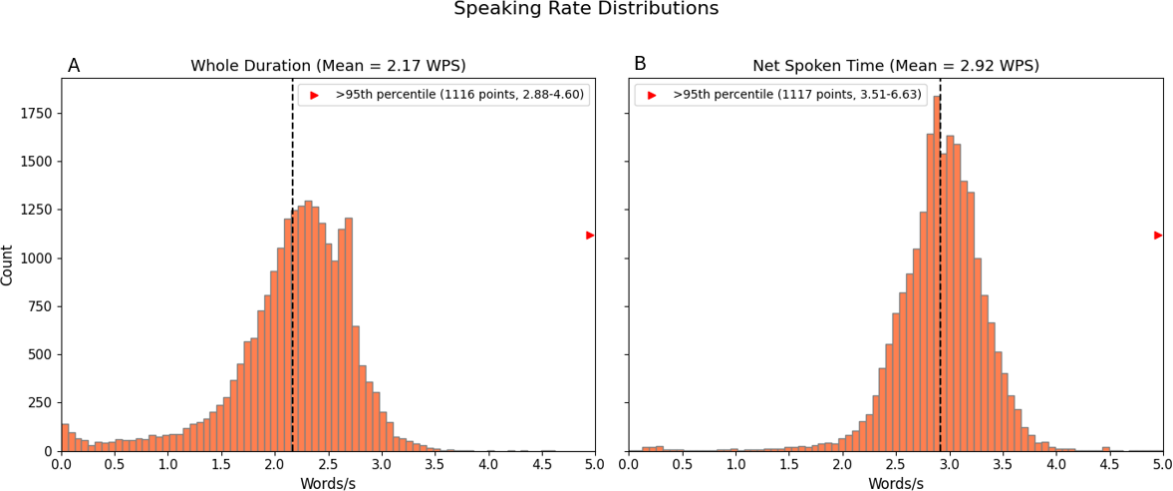
Distributions of average speaking rates extracted from transcripts with 2 methods: (**A) **Average number of words per second (WPS) over the whole session (including silences). (**B) **Average number of words per second for the net spoken time (without silences between segments). The red arrow’s height represents the number of values exceeding the 95th percentile.

Speaking rate ranged from 0 to 4.4 WPS with an average of 2.17. The net speaking rate ranged from 0.04 to 6.63 WPS with an average of 2.9 WPS (Figure 4B). Both averages fall within the reported range of the American average speaking rate in conversation [41] (1.85‐4.86 WPS).

The highest speaking rate was observed in a short transcript of an automatic answering machine. Only 30% (n=9) of transcripts with speaking rate above 3.5 WPS (Figure 4A) were longer than 20 minutes, suggesting that high speaking rate often reflected accidental recordings. For instance, of the 9 transcripts with rates greater than 4 WPS, 8 were identified as automatic voice answering machines.

Examining the ratio of overall session time to speech duration, or equivalently the ratio of speaking rate (A) to net speaking rate (B), shows that transcripts with high values (where total conversation time far exceeds spoken time) often reflect timestamp errors or short transcripts with very few words. In either case, such transcripts are not suitable for further analysis.

#### Frequent Noun Words

After filtering words according to Multimedia Appendix 1 , we identified 8,393,775 nouns in the clients’ speech and 32,591 nouns in the therapists’ speech. Multimedia Appendix 4 displays the 60 most common words before and after filtering. Therapists’ 5 most common words were “thing,” “time,” “people,” “know,” and “way,” and clients’ most common nouns were similar: “thing,” “time,” “know,” “date,” and “lot” followed by “people.” Among the 15 most common words for clients were also “friend,” “mom,” “work,” “talk,” and “life,” and therapists shared some of these nouns (“work,” “talk,” and “date”) but also frequently used “help” and “guy.”

### Classification

#### Zero-Shot Prompting

Of 850 transcripts that were given to the LLM, it identified 737 as sessions (86.7%, n=737), including 56 transcripts classified as couple’s sessions. The remaining 113 transcripts were classified as non-sessions (13.3%, n=113). The model’s certainty ratings were skewed toward the higher end of the scale, with 55 transcripts rated at 4 (7.9%) and 645 transcripts rated at 5 (92.1%, highest certainty).

#### Validation and Interrater Reliability

**Figure 5.**
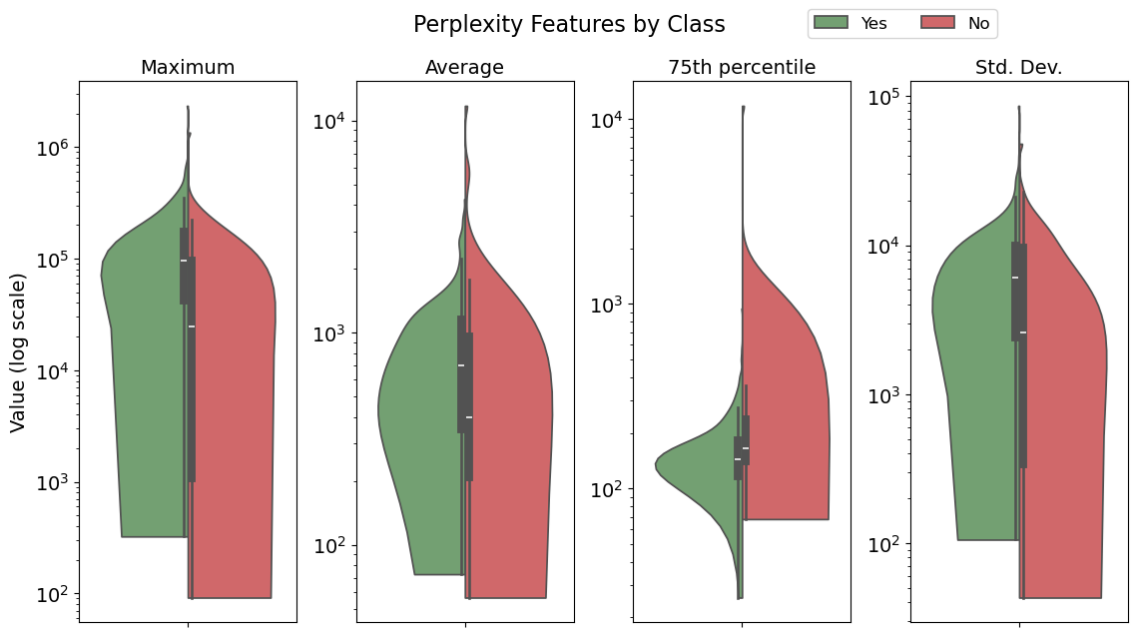
Results of permutation tests between distributions of perplexity metrics of sessions (“Yes”) and non-sessions (“No”). Perplexity metrics—maximum, average, 75th percentile, and standard deviation—were calculated over the distribution of segment perplexity for each transcript. The 75th percentile showed a significant result (mean difference = −258, *P*=.01) with sessions having lower values, while the maximum perplexity was higher for sessions (mean difference =73,888 , *P*=.007).

The raters agreed on 86.3% (44 of 51; see Figure 5A in Multimedia Appendix 5) of the test set transcripts, predominantly classifying transcripts as therapy sessions rather than nontherapy sessions (Rater 1: 61%; Rater 2: 63%). Cohen kappa score was 0.71, indicating substantial agreement [53]. Disagreements primarily revolved around identifying the session type—distinguishing between individual, couple, or family therapy—rather than determining whether a therapy session took place at all. In 43% (3 of 7) of the disagreement cases, both raters classified the transcript as a therapy session but differed on the type. In the remaining 57% (n=4), the disagreement was about whether the conversation was professional or casual. The full distribution of classifications by the raters and the model is shown in Multimedia Appendix 5.

### LLM Versus Raters

Unlike the raters, who were instructed to mark non-individual sessions (eg, family or couple therapy) as non-sessions, the LLM was instructed to identify any kind of treatment session, including family and couple therapy. This difference naturally led to disagreements between raters and the model. However, when the model explicitly identified the session type (eg, family or couples) and indicated it clearly in its explanation or summary, we considered the classification an agreement. This verification process can be automated by searching for specific keywords (eg, “couple session”) in the summary text or by using another language model to interpret the model’s output. Table 2 shows the distribution of rater-model agreement for sessions and non-sessions.

**Table 2.** Agreement between the model and the raters for transcripts agreed over by raters^a^.

	Raters: yes, n	Raters: no, n	Total, n
Model: yes, n	26	7	33
Model: no, n	2	9	11
Total, n	28	16	44

a The table features the number of transcripts in each category.

Overall, the model identified 57 sessions as couples or family therapy, demonstrating its ability not only to detect therapy sessions but also to categorize them by type.

Among the 9 disagreements between the model and the human raters, most involved the model misclassifying case management conversations as therapy. Conversely, in cases where the model misidentified conversations tagged as therapy sessions by human raters, its reasoning was because of “lack of therapeutic techniques.”

### Perplexity of Different Classes

Figure 5 shows the distributions of different statistics of perplexity of transcripts for different classes.

Among the transcripts with high perplexities, 1 transcript had exceptionally high values (mean perplexity of more than 11,000, which was approximately 11 standard deviations more than the average mean perplexity). This 4-segment transcript contained nonwords and backchannels that seemed to be transcribed noises (mostly the sound “Mhm”). Another high-perplexity file was a recorded rap song.

Of the 4 statistics—max, mean, standard deviation, and 75th percentile—a permutation test for means showed a significant result for the 75th percentile (*P*=.01), with sessions having a lower mean 75th percentile than non-sessions (mean difference=−258). In contrast, the permutation test showed that the sessions group had higher maximal values (*P*=.007).

**Figure 6.**
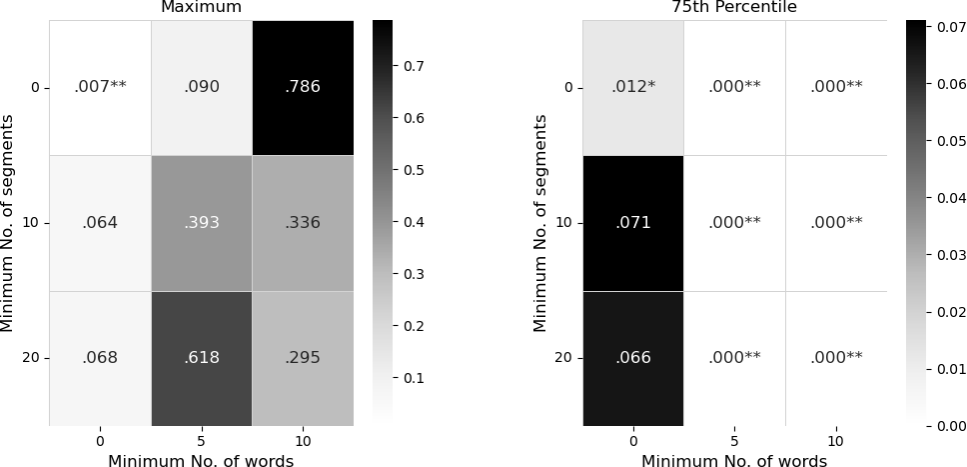
*P* values of permutation tests comparing sessions and non-sessions perplexity measures across parameter settings—minimum number of segments and minimal number of words per segment. (**A) **Maximum perplexity shows no significant values across parameters except when no restrictions are applied and (**B) **75th percentile of perplexity is significant for most parameter combinations. Bright colors indicate lower *P* values, *P<.05, **P<.01.

To check the robustness of these results, we repeated the test after limiting the calculation to segments with minimal number of words (minimum words per segment [MWPS]=5, 10) and omitting transcripts with fewer than a minimal number of relevant segments (minimal number of segments [MS]=10, 20). These tests showed that the 75th percentile measure (Figure 6A) remained significant under these parameter changes, whereas the max perplexity measure (Figure 6B) lost significance when limitations were applied (MS>0 or MWPS >0).

Furthermore, we evaluated the classification performance of a 75th percentile perplexity–based classifier by analyzing receiver operating characteristic (ROC) curves and precision-recall metrics across parameters (MWPS, MS). To align with perplexity values, in this analysis, higher perplexity values correspond to the positive class—non-sessions—while sessions constitute the negative class. The optimal threshold was determined by maximizing the difference between true positive rate and false positive rate, prioritizing accurate classification of non-sessions. The results (Table 3) indicate moderate discriminative ability, with ROC area under the curve values ranging from approximately 0.62 to 0.73 and precision increasing alongside minimal number of segments. Precision tends to increase with larger segment sizes (up to ~0.63), while recall decreases (~0.26).

**Table 3.** Classification performance of the 75th percentile perplexity–based filter across varying thresholds for minimal words per segment (MWPS) and minimal number of segments (MS).

MWPS	MS	Sessions	Non-sessions	ROC AUC	PR AUC	Precision	Recall
0	0	285	50	0.618	0.240	0.200	0.740
0	5	285	43	0.636	0.198	0.182	0.767
0	10	284	42	0.633	0.196	0.178	0.762
5	0	285	47	0.664	0.354	0.353	0.255
5	5	284	41	0.657	0.331	0.333	0.268
5	10	284	40	0.665	0.335	0.333	0.275
10	0	285	44	0.687	0.378	0.625	0.227
10	5	284	40	0.701	0.380	0.625	0.250
10	10	284	38	0.726	0.392	0.625	0.263

a The table reports the number of transcripts remaining in each class after filtering outliers, the receiver operating characteristic area under the curve (ROC AUC), precision-recall AUC (PR AUC), precision, recall, and F1 scores. Increasing the minimal words and segments thresholds improves ROC AUC and precision but reduces recall.

## Discussion

### Principal Findings

With a dataset of over 22,000 unfiltered transcripts of recordings associated with behavioral treatment sessions, our primary objective in this methodological paper was to illustrate a systematic approach for characterizing the data and implementing a filtering process for subsequent analysis in academic research. Prior research on leveraging machine learning and natural language processing methods for classifying large text datasets, both conversational [54] and nonconversational [55,56], has focused mostly on content-based classification rather than the contextual framework of conversations. Our proposed methodology integrated human evaluation, statistical analysis, and automated tools, including LLMs, emphasizing the importance of contextual and relational features, which are especially critical for distinguishing therapy sessions from non-sessions.

The preliminary analysis identified a significant proportion of non-session transcripts within the dataset. These non-sessions encompassed brief case management encounters, informal conversations, mock sessions, and incidental recordings captured between sessions. Additionally, some transcripts included errors such as incorrect speaker identification, text duplication, and incomprehensible content [25]. These findings highlight the necessity for systematic filtering mechanisms to exclude low-quality and non-session data. Given that only approximately half of the dataset was found suitable for analysis, leveraging automated classification and quality scoring can significantly enhance the dataset’s utility for research purposes.

Recent works have listed features for characterizing texts in corpora [35,57]. In this paper, we have focused on 3 basic ways to extract relevant statistics and examined if outliers could be indicative of problematic transcripts:

Conversation length—Analysis of conversation length distributions revealed that shorter transcripts often represented non-sessions, such as case management encounters, as expected for this type of service, phone calls, or noise. In contrast, unexpectedly long conversations may result from sessions being concatenated or recordings extending beyond the session’s actual duration, thus requiring careful preprocessing.

Speaking rate—Speaking rate has been evaluated in many contexts, and it has been shown that its values can predict speakers’ features [41], detect speech anomalies, and identify change in conversation dynamics or context [42]. We found that high speaking rate was associated with shorter transcripts, particularly erroneous recordings such as answering machine messages. This finding supports the use of speaking rate as a potential indicator of non-sessions. Additionally, the ratio between speech duration and overall recording duration may serve as a marker for time-labeling errors.

Content analysis—Bag-of-Words is a basic tool for evaluating semantic content. Through word count (mostly nouns), themes and topics can be discovered and ultimately enhance text classification [58-60]. In this paper, we used it as a “reality check” to reveal the contents of the transcripts at hand. Our content analysis provided insights into the vocabulary and topics typical of therapeutic conversations. The most prevalent words were related to everyday life (“relationship,” “house,” “job,” “today,” “school,” “sleep,” and “car”), clients' inner world (“thought,” “want,” “feel,” and “need”), resolving issues (“situation,” “problem,” “help,” and “care”), and relationships (“kid,” “love,” “date,” “sister,” “couple,” “dad,” and “guy”), emphasizing the centrality of these themes in these conversations. Comparing individual transcript themes against the topics that were found in this analysis could be an indicator for being a genuine session. However, it is of note that this analysis ignores the context in which words appear.

#### Perplexity Analysis

Perplexity is traditionally used to evaluate language models [61,62]. In this study, however, we used it to evaluate the comprehensibility and uniqueness of text segments [63,64] in order to gain insight into potential differences between sessions and non-sessions. Transcripts with higher perplexity scores often contained transcription errors or nonverbal content (eg, noise and backchannels).

Sessions initially exhibited higher maximal transcript perplexity than non-sessions; however, this result was no longer significant when outliers, namely, transcripts with few segments or short segments, were omitted. This suggests that the higher maximal perplexity values in sessions are not necessarily indicative of more verbally complex content but rather stem from transcription errors, backchanneling, discourse markers, or interrupted recordings, which tend to produce short segments and short transcripts. These findings indicate that sessions, in general, do not have higher maximal perplexity than non-sessions. This interpretation is further supported by the finding that the 75th percentile perplexity score for sessions was lower than non-sessions after removing outliers. This may suggest that sessions contain more structured and predictable language, particularly when compared to background noise or accidentally captured conversation fragments. This distinction is particularly useful because it suggests that 75th percentile perplexity could serve as a reliable feature in a non-session filtering model. Unlike maximal perplexity, which was influenced by errors and outliers, the 75th percentile measure remains stable across different parameter settings, reinforcing its robustness as an indicator of session-like structure.

To support this claim and further evaluate its ability to differentiate sessions from non-sessions, we assessed a 75th percentile perplexity–based classifier across varying thresholds for MS and MWPS. The optimal threshold remained consistent across conditions, suggesting its practical applicability as a filtering criterion. However, the results indicate moderate-to-low discriminative performance, with precision improving under stricter outlier exclusion. For instance, removing transcripts with fewer than 10 segments of more than 10 words each yielded the highest precision (~0.63) but substantially reduced recall (~0.26). This pattern likely reflects the dataset imbalance—where non-sessions are the minority—and the observation that outliers were predominantly non-sessions, suggesting they tend to exhibit relatively high 75th percentile perplexity. These findings reinforce the conclusion that this metric is particularly useful when filtering aims to prioritize precision, though caution is needed due to the class imbalance impacting these metrics.

Overall, our results suggest that perplexity can be used to identify flawed, incomprehensible, or highly improbable text segments and may serve as a useful tool for detecting low-quality or non-session transcripts, but not as a standalone classifier. To validate our results and extend this work, we propose 2 key next steps: (1) to distinguish nonprofessional conversations from flawed transcripts to determine which group is more clearly separable from sessions through perplexity measures. This distinction is critical for understanding the sources of high- and low-perplexity segments. (2) To replicate these analyses with a larger set of labeled transcripts to overcome data imbalance.

#### Large Language Model Classification

Research on LLMs has explored their use for both text classification and mental health text analysis as separate tasks. Prior work has demonstrated these models’ ability to extract key concepts, analyze text dynamics, and identify psychological concepts [14,65-69]. In this study, we integrated both tasks, leveraging zero-shot prompting to analyze mental health conversations with the goal of classification. Our findings indicate that with zero-shot prompting, the model can classify transcripts effectively, showing high agreement with human coders while demonstrating robustness in handling transcript errors. For example, even when speaker identification referenced only a therapist and a patient, the model correctly identified couple or family sessions by interpreting contextual and semantic cues. Additionally, the LLM successfully corrected speaker identification errors, highlighting its potential as an automated error-correction tool.

Instances of disagreement between human coders and the LLM often stemmed from the model’s sensitivity to therapeutic techniques. One of the classification guidelines was the presence of such techniques, and the model appeared to weigh them heavily when the conversational framing was ambiguous. Furthermore, in some cases, we found discrepancies between the binary classification and the LLM’s explanation. For instance, despite classifying a conversation as a session, the model noted: *The therapist and psychologist discuss updates on multiple client cases, including challenges with clients’ behaviors*, recognizing both speakers as therapists. This example underscores the importance of including model explanations as part of the classification process. Future work should investigate these discrepancies and refine classification guidelines accordingly.

Finally, while research on LLMs’ ability to analyze conversations through prompting is still evolving, existing studies have yielded inconsistent findings regarding their effectiveness in analyzing conversational contexts [14,65-69]. Our results suggest that when provided with full conversation transcripts, LLMs can capture nuanced textual and relational dynamics, offering valuable insights into participants’ interactions. While recent studies have used these capabilities in text-based applications, we demonstrated their applicability to conversations, where relational and dynamic elements are crucial in distinguishing sessions from non-sessions. Thus, although few-shot learning or fine-tuning may further enhance classification accuracy [55,56], our findings suggest that these techniques may not be strictly necessary for effective classification.

### Limitations

Defining when a transcript reflects a treatment session presents a challenge: sessions share many aspects with nontherapeutic conversations, and some therapeutic techniques might appear as trivial exchanges. In this paper, we proposed both statistical heuristics and explicit guidelines to evaluate conversations in light of this question.

While we offer a methodological examination of a transcript dataset and highlight considerations for careful filtering, defining a filtering model is beyond the scope of this study. Since ground-truth labels were unavailable for supervised classification, we address this by identifying features that provide valuable insights into the classification process and by demonstrating how LLMs can assist experts when guided by well-designed prompt engineering. Hence, the approach presented here may serve as a foundation for developing a comprehensive classification model.

This study delineates a general framework and provides guidelines for working with conversational datasets in the absence of prior knowledge or structured labels. However, variation exists across datasets in format, content, quality, and the proportion of non-session files and transcription errors. While this paper applies its suggested framework on a large and varied dataset, it still features working with a single data platform. Variation in platforms and datasets calls for researchers to (1) detect any peculiarities relevant to their datasets and acknowledge them before referring to the relevant steps in the framework and (2) cultivate content matter expertise for datasets they analyze or better—conduct studies in multidisciplinary teams and keep a human (expert) in the loop.

### Future Research

Our findings suggest that while statistical features can provide broad insights into conversational structure and flag irregularities that may correlate with non-sessions, LLMs add a complementary layer by capturing the essence, dynamics, and nature of conversations. As a proof of concept, this study demonstrates the potential of LLM-based classification; however, further validation, including human annotation on larger samples, is needed. In a treatment setting, we envision a semiautomated pipeline that integrates statistical and semantic features with LLMs. Transcripts would first be screened using metrics such as perplexity, duration, and speaking rate; those within a normal range would then be analyzed by an LLM to classify the conversation type—treatment session or unrelated. Periodic human review of selected transcripts would ensure model validity, with reviewer feedback used to refine prompts or feature thresholds dynamically. This “human-in-the-loop” workflow enables continuous processing while limiting manual review to a small subset of cases. Future work should evaluate different LLM models across diverse behavioral health datasets to determine their ability to capture nuances in treatment styles.

Regarding statistical feature–based outliers filtering, additional research could enhance its efficiency and interpretability. Incorporating more informative linguistic features—such as the frequency of backchanneling cues, discourse markers, and silent pauses—into interpretable models such as decision trees may improve classification while remaining computationally efficient compared to fine-tuning large-scale models. Moreover, content analysis could reveal how the themes extracted from an individual session differ from those found across the dataset, highlighting deviations that might indicate non-session transcripts. Examining bigrams and multi-word expressions could also refine differentiation between informal conversations and structured therapeutic sessions, while helping to detect common transcription errors, such as misinterpretations caused by background noise.

Perplexity analyses could examine the relationship between different segment error types, such as misspelling, and perplexity by categorizing segments accordingly. Additionally, applying a mixed-model analysis to account for the statistical dependencies of perplexities within individual transcripts. Moreover, the interaction between perplexity and factors such as speaker familiarity or amorphous nature of the conversation remains an open question and could help explain cases where high perplexity signals meaningful conversational ambiguity rather than transcription errors. Finally, improving transcript quality remains a critical avenue for future research. Existing methods for correcting speaker diarization errors and ASR mistakes without access to the original audio [70,71] could be integrated into the preprocessing pipeline to enhance transcript reliability before filtering. Implementing these techniques could enhance the preprocessing stage, improve the accuracy of extracted features, and ultimately enhance the filtering process and its success rates.

### Conclusion

This study demonstrated the importance of integrating human judgment with automated tools when processing large, unstructured datasets. We assess secondary data—data collected independently of current research—where initial human evaluation is critical for understanding dataset characteristics such as readability, content, and diarization quality. This foundational knowledge can inform the development of effective filtering strategies. While basic statistics, perplexity, and LLM prompting facilitate automated filtering, preliminary human review remains essential for understanding dataset variability and refining classification features. This hybrid approach ensures adaptable and accurate filtering processes, even in the presence of transcription errors.

## Supplementary material

10.2196/78082 Multimedia Appendix 1 Clean-up words.

10.2196/78082 Multimedia Appendix 2 Amazon Bedrock models prices.

10.2196/78082 Multimedia Appendix 3 Prompt.

10.2196/78082 Multimedia Appendix 4 Most common words: before and after filtering.

10.2196/78082 Multimedia Appendix 5 Session versus non-session classification results: (A) inter-rater answers distribution and (B) distribution of agreement between the model and the human raters.

## Abbreviations

AI: artificial intelligence
ASR: automatic speech recognition
LLM: large language model
MS: minimal number of segments
MWPS: minimum words per segment
ROC: receiver operating characteristic
WPS: words per second
