# Supplementary figures and images for: Preprocessing Large-Scale Conversational Datasets: A Framework and Its Application to Behavioral Health Transcripts
Source: JMIR Form Res. 2025 Oct 24;9:e78082. doi: 10.2196/78082 (PMC12551936; doi:10.2196/78082)

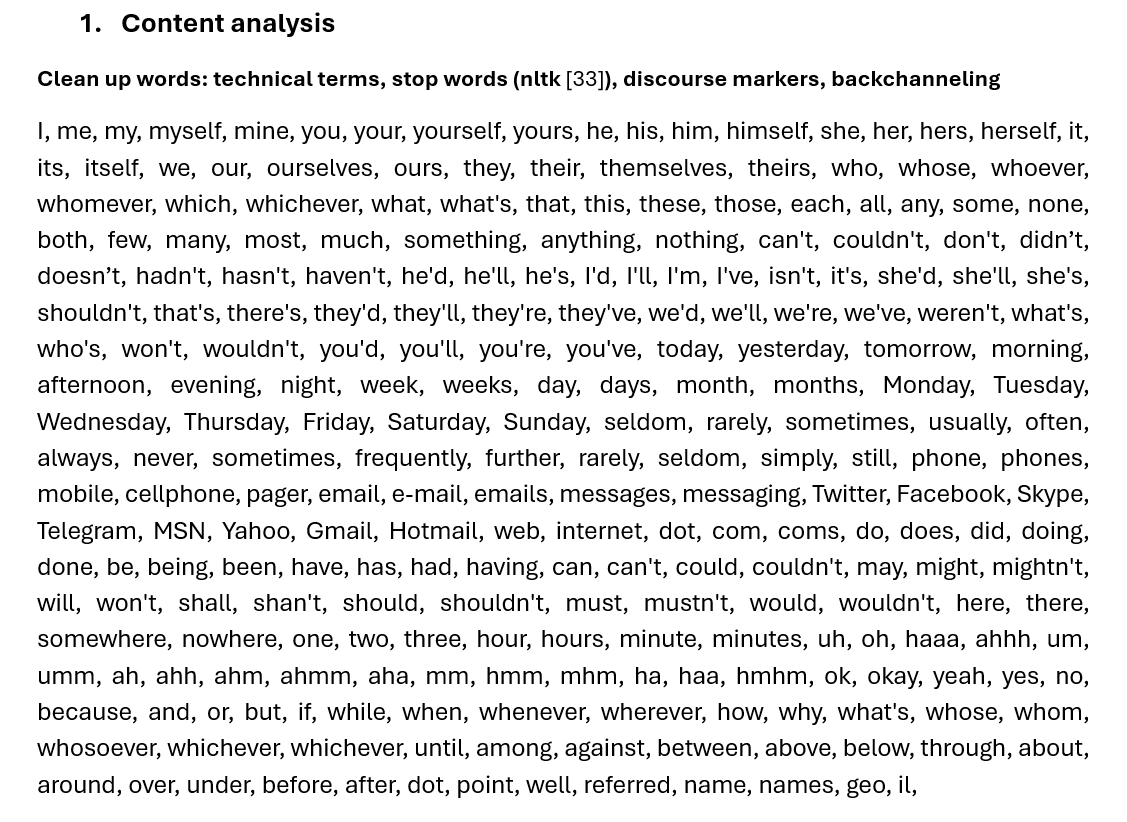

Supplement: Multimedia Appendix 1 [file formative-v9-e78082-s001.png]

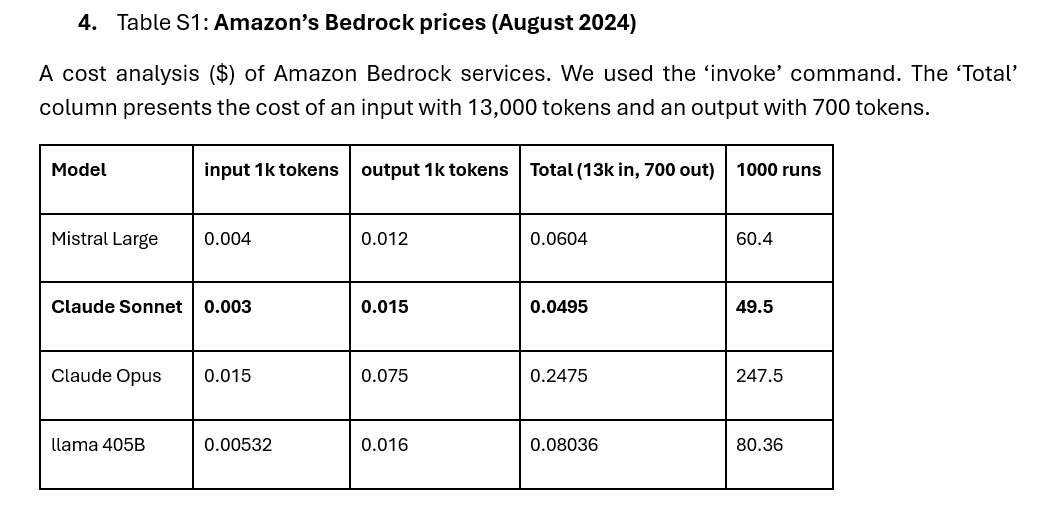

Supplement: Multimedia Appendix 2 [file formative-v9-e78082-s002.png]

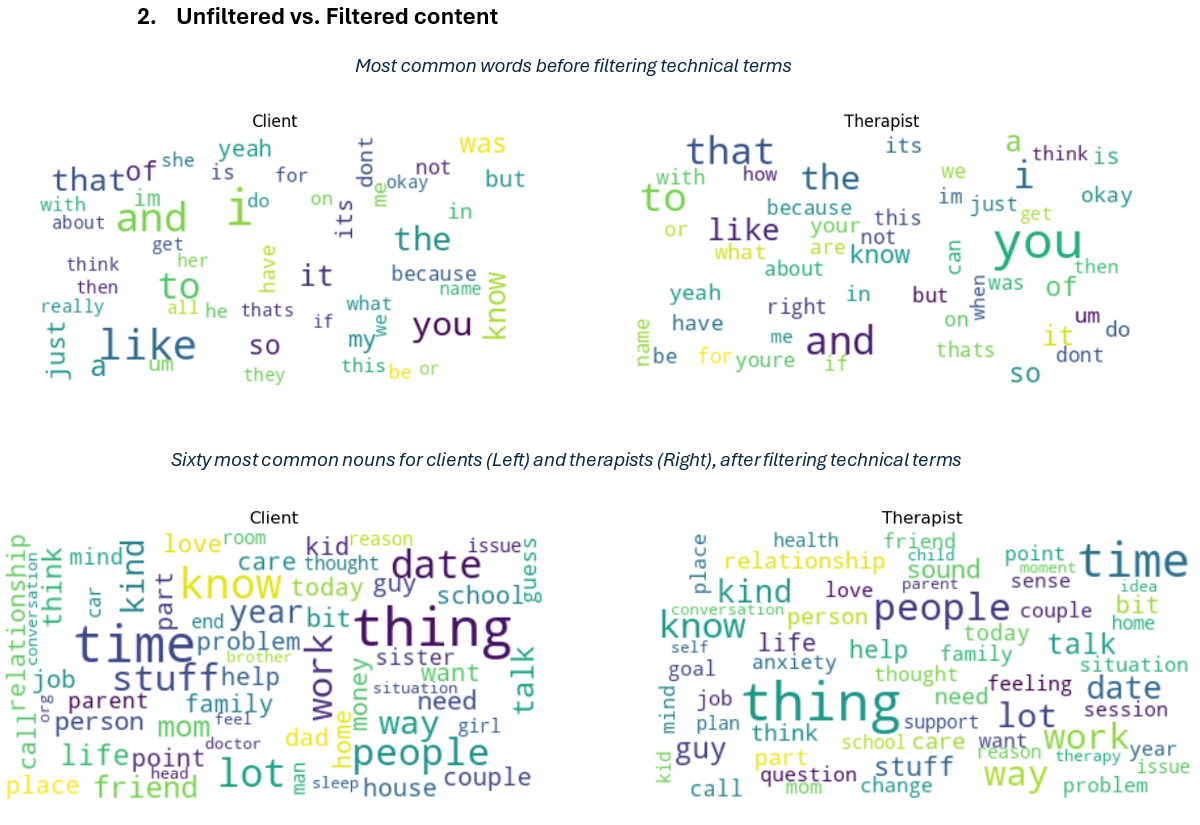

Supplement: Multimedia Appendix 4 [file formative-v9-e78082-s004.png]

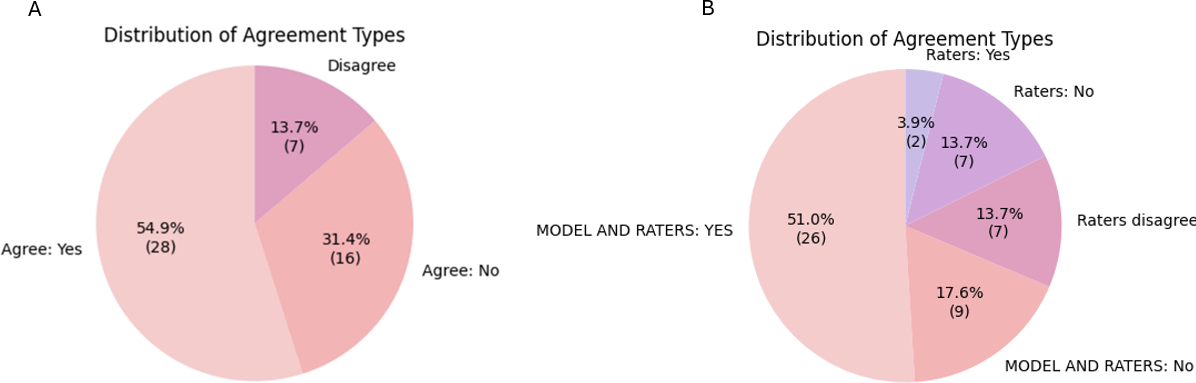

Supplement: Multimedia Appendix 5 [file formative-v9-e78082-s005.png]
