# Supplementary material for: Preprocessing Large-Scale Conversational Datasets: A Framework and Its Application to Behavioral Health Transcripts
Source: JMIR Form Res. 2025 Oct 24;9:e78082. doi: 10.2196/78082 (PMC12551936; doi:10.2196/78082)
Supplement: Multimedia Appendix 3 [file formative-v9-e78082-s003.docx]

1. **Prompt**

<description>

As a clinical psychologist, your task is to determine whether the conversation

 in the brackets is a behavioral treatment session or not.

Please provide a concise report (up to 200 words) explaining your decision, and

rate your certainty on a scale of 1-5 (1 being least certain, 5

being most certain), add a two-sentence summary of the content of the

conversation.

The report must follow the following format:

   <decision>decision (yes/no): (provide only "yes" or "no")</decision>

   <certainty_rate>certainty rate: (provide a number between 1-5)</certainty_rate>

   <explanation>explanation: (provide your explanation here)</explanation>

   <summary>summary: (provide your summary here)</summary>

</description>

<guidlines>

   <item>1. Conversation structure:

   - Is there a clear back-and-forth between two individuals?

   - Does one person primarily share personal experiences while the other listens and responds?</item>

   <item>2. Content:

   - Does the conversation focus on personal matters, emotions, behaviors, or experiences?

   - Are there discussions about goals, progress, or changes in thoughts or behaviors?</item>

   <item>3. Therapeutic elements:

   - Does one speaker demonstrate active listening, empathy, and respectful responses?

   - Are there instances of therapeutic techniques: reframing, challenging thoughts, providing coping strategies, helping with problem solving, practicing interpersonal skills, etc.?</item>

   <item>4. Professional language:

   - Does one speaker use terminology or techniques associated with behavioral therapy?

   - Are there references to previous sessions, homework, or treatment plans?</item>

   <item>5. Context clues:

   - Are there mentions of confidentiality, session time limits, or scheduling future appointments?</item>

   Please note any other relevant factors that influenced your decision. If the conversation appears to be a different type of interaction (e.g., podcast, sales call), briefly explain why it doesn't qualify as a behavioral treatment session.

   Here is the conversation:

</guidelines>
